# Telomere length dynamics in human memory T cells specific for viruses causing acute or latent infections

**DOI:** 10.1186/1742-4933-10-37

**Published:** 2013-08-26

**Authors:** Joel M O'Bryan, Marcia Woda, Mary Co, Anuja Mathew, Alan L Rothman

**Affiliations:** 1 Division of Infectious Diseases and Immunology, University of Massachusetts, Medical School, Worcester MA, USA; 2 Institute for Immunology and Informatics, University of Rhode Island, Providence, RI, USA

**Keywords:** Ageing, Telomere, T cell memory, CD45RA, FlowFISH, Influenza A virus, Cytomegalovirus, Vaccinia virus, Varicella zoster virus, BrdU labeling

## Abstract

**Background:**

Declining telomere length (TL) is associated with T cell senescence. While TL in naïve and memory T cells declines with increasing age, there is limited data on TL dynamics in virus-specific memory CD4^+^ T cells in healthy adults. We combined BrdU-labeling of virus-stimulated T cells followed with flow cytometry-fluorescent in situ hybridization for TL determination. We analyzed TL in T cells specific for several virus infections: non-recurring acute (vaccinia virus, VACV), recurring-acute (influenza A virus, IAV), and reactivating viruses (varicella-zoster virus, VZV, and cytomegalovirus, CMV) in 10 healthy subjects. Additionally, five subjects provided multiple blood samples separated by up to 10 years.

**Results:**

VACV- and CMV-specific T cells had longer average TL than IAV-specific CD4^+^ T cells. Although most virus-specific cells were CD45RA^-^, we observed a minor population of BrdU+ CD45RA^+^ T cells characterized by long telomeres. Longitudinal analysis demonstrated a slow decline in average TL in virus-specific T cells. However, in one subject, VZV reactivation led to an increase in average TL in VZV-specific memory T cells, suggesting a conversion of longer TL cells from the naïve T cell repertoire.

**Conclusions:**

TLs in memory CD4^+^ T cells in otherwise healthy adults are heterogeneous and follow distinct virus-specific kinetics. These findings suggests that the distribution of TL and the creation and maintenance of long TL memory T cells could be important for the persistence of long-lived T cell memory.

## Background

Virus-specific T cell proliferative responses are detectable for decades after the initial infection [1-3] but how this T cell memory is established and maintained is not clear. Telomere length (TL) has been shown to be a critical determinant of T cell replicative capacity and in vivo persistence in humans; clinical trials have clearly shown that adoptive transfer of minimally-expanded tumor-infiltrating lymphocytes with long telomeres correlated with better in vivo persistence and proliferation, while excessive in vitro expansion prior to adoptive transfer lead to shortened telomeres, and correlated with poor in vivo persistence [4,5].

Low frequencies of virus-specific T cells and the limited number of known virus epitopes has restricted the ex vivo study of TL mainly to CD8^+^ T cells specific for a few immunodominant epitopes with more limited studies of CD4^+^ T cells [6-9]. Despite robust CD8^+^ T cell responses during a primary infection, CD4^+^ memory T cell responses have been reported in some studies as more durable than CD8^+^ T cell responses [2,10-12]. Differing abilities of CD4^+^ and CD8^+^ T cells to up-regulate telomerase during activation and thus enhance telomere maintenance during this activation-induced proliferative phase have been proposed to account for these differences [13].

Memory T cells turnover in vivo more rapidly than naïve T cells [14-16]. However, T cells undergoing antigen-independent homeostatic proliferation do not express the high levels of telomerase necessary to prevent replication-driven TL erosion [17-19]. This increased memory T cell turnover and lack of telomerase should theoretically lead to the senescence and loss of the proliferative capacity of memory T cells within a decade. On the other hand, periodic antigen-driven reactivation of memory T cells specific for recurrent or latent infections could drive additional rounds of proliferation with expression of telomerase to explain their continuing persistence [17,20], although other data suggest that telomerase expression declines with each round of activation [19]. But this explanation fails in the face of reports of detectable poxvirus-specific T cells 5 to 6 decades after a smallpox vaccination, a situation where there are no recurring exposures [2]. Recently, the demonstration in humans and non-human primates of the presence of a T cell memory subset with stem cell-like renewal properties, termed T_SCM_ cells, may provide a basis for the persistence of very long-lived T cell memory [21,22]. A substantially increased TL in this memory subset, relative to more differentiated memory T cells, could provide a mechanistic explanation for the in vivo longevity of the T_SCM_ cells.

Virus-specific memory T cells are maintained in vivo under diverse conditions. Two common latent-reactivating herpesviruses differ in their host interactions; cytomegalovirus (CMV) is thought to establish latency in a wide range of tissues and cell types, especially myeloid cells, and may reactivate frequently, whereas varicella-zoster virus (VZV) establishes latency only in ganglionic neurons and reactivates infrequently [23,24]. Nevertheless, both require life-long T cell-mediated immunity for their control within latently infected hosts [25,26]. Patterns of antigen exposure also differ for acute viral infections, such as influenza A virus (IAV), to which humans are likely repeatedly (seasonally) exposed, and vaccinia virus (VACV), a poxvirus with a quite limited exposure outside of the controlled vaccination setting. We used in vitro BrdU labeling to detect virus-specific memory T cells based on proliferation in response to virus stimulation, and then used flow cytometry fluorescence in situ hybridization (flowFISH) to measure TLs in virus-specific (BrdU+) cells. Importantly, flowFISH allows for the analysis of individual cells and can be multiplexed with (a limited number of) cell phenotypic markers such as CD45RA [27].

We first developed and validated a modified flowFISH TL assay on in vitro-expanded T cells by comparison with Southern blotting telomere restriction fragment length (TRF) results. We then performed a cross-sectional study of 10 healthy adults, with 5 of these subjects providing sequential samples which allowed for a longitudinal study of T cell TLs. Our results reveal diverse effects of virus re-infection and reactivation on T cell TL and the maintenance of T cell memory.

## Results

### FlowFISH analysis of T cell telomere length in in vitro-expanded T cells

**Figure 1:**
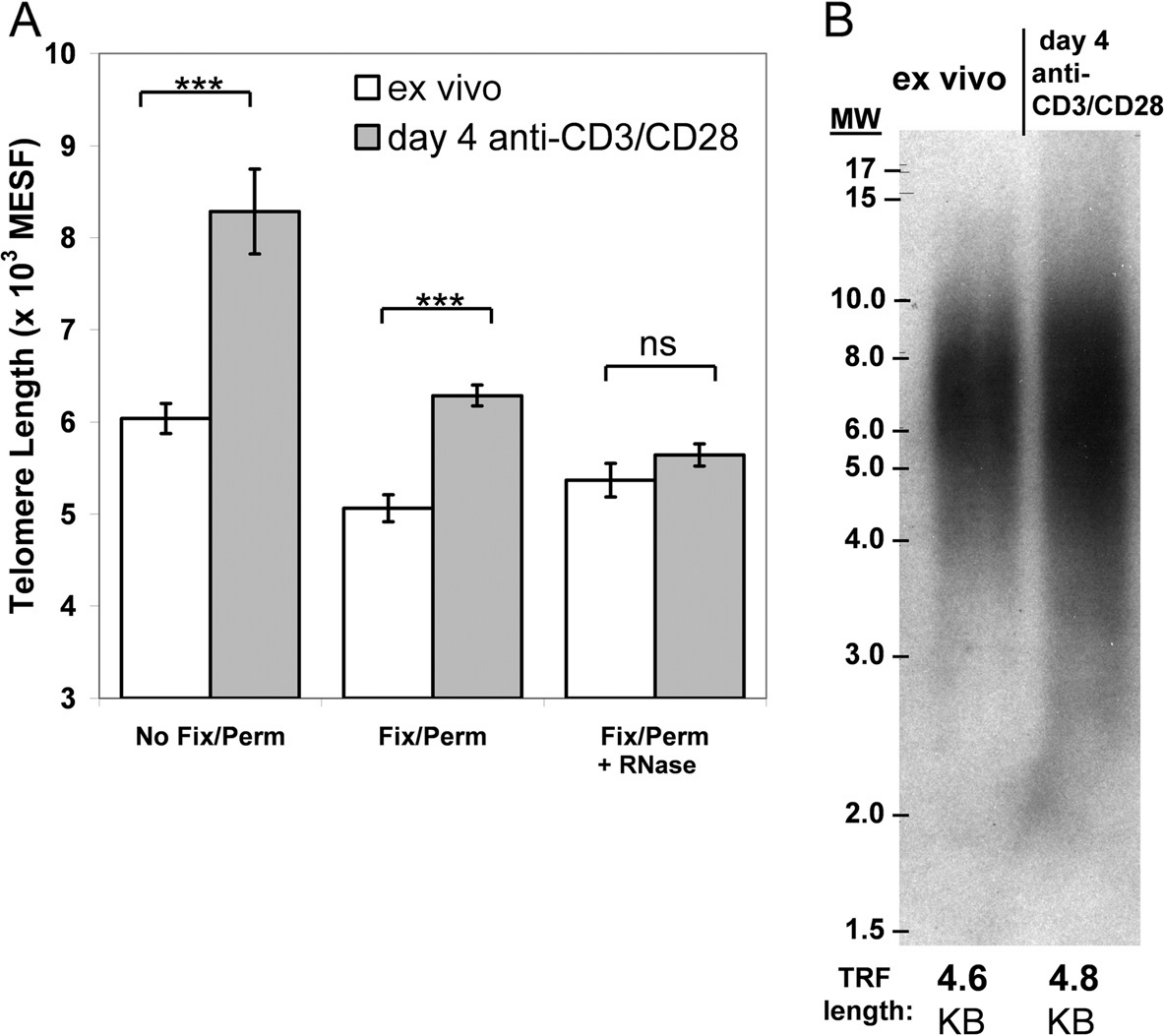
**Telomere length (TL) measurement using flowFISH on proliferating T lymphocytes depends on fixation-permeabilization and RNA nuclease treatment.** PBMC from healthy adults were either stimulated with immobilized anti-CD3 and anti-CD28 for 4 days or were cryopreserved on day 0 and thawed for processing on day 4; CD3^+^ T cells were magnetically sorted from both samples on day 4. Each sample was divided for flowFISH TL analysis and telomere restriction fragment (TRF) Southern blotting. **(A)** FlowFISH analysis using 3 different pre-hybridization conditions: without fixation-permeabilization prior to probe hybridization (no fix/perm), with fixation-permeabilization (Fix/Perm) prior to hybridization, and with fixation-permeabilization followed by RNase treatment prior to hybridization (Fix/Perm +RNase). Statistical comparisons were done using unpaired *t*-test, *** p<0.001, and ns=not significantly different. **(B)** TRF Southern blot analysis. DNA was extracted from CD3+ T cells isolated ex vivo or after stimulation with anti CD3+CD28 for four days and subject to Southern Blot Analysis. TRF lengths are shown at the bottom of both lanes and are the average of three separate 20 pixel-wide analyses using MatLab software running the MaTelo macro (see Methods).

We sought to compare TL in memory T cells in healthy adults specific for viruses causing acute (VACV, IAV) versus latent (VZV, CMV) infections. Since the frequency of virus-specific memory T cells in PBMC is generally too low in healthy humans for a robust TL analysis directly ex vivo, we expanded PBMC in vitro with viral antigens. To determine how this in vitro expansion may have affected TL, we compared TL in total T cells isolated from fresh PBMC to in vitro-expanded T cells using flowFISH [28]. Initial flowFISH experiments revealed inflated TL measurements in the in vitro expanded T cells (p<0.001, Figure 1A left bars). However, these flowFISH-derived TL estimates differed from TRF Southern blotting results (compare Figure 1B with Figure 1A left bars), where the TRF results were similar for the expanded T cells and those analyzed ex vivo. This suggested that the inflated flowFISH TL estimate was an assay artifact.

We therefore tested several modifications to the flowFISH protocol. Inclusion of a pre-hybridization fixation-permeabilization step substantially reduced the inflated telomere probe fluorescence, resulting in mean TL estimates closer to the TRF results (Figure 1A middle bars). We further added an RNase treatment step [29], in light of reports that telomeres are transcribed and thus providing additional targets for binding of the flowFISH telomere peptide nucleic acid (PNA) probe [30,31]. We optimized the RNase concentration and treatment times using a separate PNA probe to the 7SK small nuclear RNA [32] to ensure this protocol provided nuclease access to and digestion of nuclear-localized RNAs (data not shown). The combination of these steps gave minimized background fluorescence for the Cy-5 labeled telomere probe (Additional 1: Figure S1) and produced flowFISH TL estimates in agreement with the TRF results (Figure 1A right bars).

### TL measurement in T lymphocytes that proliferate to viral antigens

**Figure 2:**
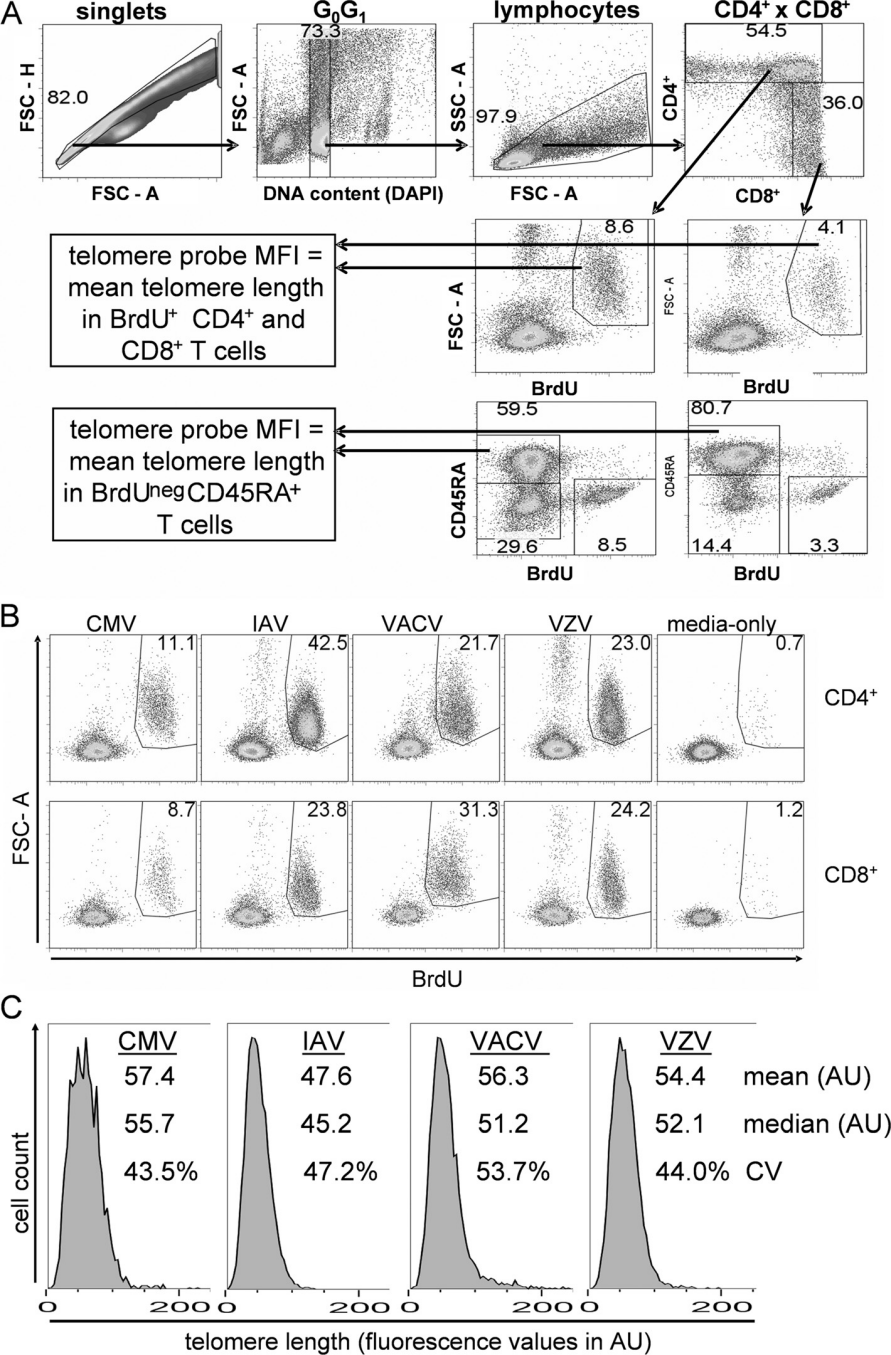
**BrdU-flowFISH allows for TL measurement in proliferating CD4**^**+ **^**and CD8**^**+ **^**T lymphocytes. (A)** Flow cytometry gating strategy for TL measurement from probe mean fluorescence intensity (MFI) in BrdU^+^ CD4^+^ and CD8^+^ cells and in BrdU-negative naïve (CD45RA^+^) T cells. **(B)** Representative proliferative responses (BrdU+ FSC-A^high^ population) of CD4+ and CD8+ T cells to viral antigen. Values shown are frequencies of CD4^+^ or CD8^+^ T cells that were BrdU+. **(C)** Histograms of TL distribution for virus-specific CD4^+^ T cells defined as in **B**. Mean and median fluorescence intensity values, in arbitrary units (AU), are shown for each plot. Coefficient of variation (CV) is also shown.

Flow cytometry gating allowed discrimination of CD4^+^ T cell subsets and estimation of TL in each subset (Figure 2A). Others have shown that the AlexaFluor® dyes and related organic small molecule dyes survive the in situ hybridization protocol and are useable, albeit with a somewhat reduced intensity, for flowFISH [33,34]. Although the hybridization altered the intensity of CD4^+^ and CD8^+^ staining (Figure 2A, far right panel), the two primary T cell subsets (CD4^+^CD8^-^ versus CD4^-^CD8^+^) could be reproducibly defined (Additional 1: Figure S2). To identify T cells that proliferated to virus antigen, we labeled cells with BrdU during the final 72 h of a 7 day incubation and stained with a fluorochrome-conjugated anti-BrdU antibody after telomere probe hybridization. The flowFISH protocol accommodated anti-BrdU staining as a post-hybridization step (Figure 2A).

We determined the specificity of in vitro proliferation to the viral antigens. It is difficult to identify subjects who have not been exposed to IAV or VZV. For CMV, seronegative status may not represent individuals who are truly CMV naïve by more sensitive PCR methods [35]; in contrast most have limited exposure to VACV outside of vaccination. Evidence for CMV-specific memory T cells in CMV seronegative healthy adults has recently been reported [36]. We therefore tested responses to VACV in PBMC collected from five subjects prior to initial smallpox vaccination to determine the specificity of the in vitro response and provide insight to the background levels of BrdU staining in negative controls. In these pilot studies, CD4^+^ T cells in PBMC from all subjects readily responded to IAV antigen, while responses to VACV antigen in PBMC samples prior to vaccination with VACV were close to the media-only control (Additional file 1: Figure S3). Accordingly, we used a BrdU+ T cell frequency of 5-fold or higher above the control culture of media-only background proliferation as the criterion for a positive response. CD4^+^ T cells from nine of our ten healthy adult subjects proliferated to CMV antigen by this criteria; CD4^+^ T cells from all subjects met this positive response criteria to IAV, VACV, and VZV (Table 1). CD4^+^ and CD8^+^ responses from a typical subject are shown in Figure 2B. Proliferative responses in CD8^+^ T cells in these antigen-stimulated cultures were generally lower; therefore the cross-sectional study results presented here are limited to CD4^+^ T cell responses.

**Table 1:** **CD4**^**+ **^**T cell proliferation responses in PBMC from ten healthy donors**

			Fold increase in proliferation*			
		**% proliferation**				
**Donor #**	**Age/Gender**	**Media**	**CMV**	**IAV**	**VACV**	**VZV**
1	51/M	2.1	*2.7*	23.4	13.8	8.5
2	49/M	0.6	20.0	76.6	65.2	41.3
3	41/F	0.7	7.1	40.4	5.1	73.3
4	39/M	0.5	162.8	69.4	66.8	60.2
5	49/F	0.2	24.5	146.0	75.6	122.5
6	61/F	0.4	125.5	67.3	23.3	29.3
7	43/M	0.7	16.1	64.1	19.6	58.0
8	28/F	1.4	9.1	24.1	19.3	29.4
9	26/M	0.1	92.0	96.0	230.0	283.0
10	35/M	0.5	15.4	106.0	35.8	69.0
Number of + responses			9	10	10	10

* Fold increase in proliferation is the ratio of the frequency of BrdU+ CD4+ T cells in response to virus stimulation divided by the %BrdU+ frequency in response to media.

Gender: M – male, F – female.

The use of CD45RA staining allowed for TL measurement in the mostly naïve non-proliferated (CD45RA^+^ BrdU^neg^) T cell subsets as a point of reference for each subject (Figure 2A). Although the CD45RA^+^ phenotype does not exclusively identify naïve T cells, this marker captures the naïve T cells, which typically form the majority of this phenotype in vivo in healthy young and mid-life adults. Importantly for our use here, this BrdU- CD45RA^+^ subset from the media-only control for each donor provided a TL context to allow for a point of reference for each individual’s memory TL comparisons. TL in CD45RA^+^ T cells cultured in vitro correlated well with TL in CD45RA^+^ cells ex vivo (Additional 1: Figure S4A). To evaluate the diversity in TL distribution in each sample, the single-cell flowFISH enabled an evaluation of median TL and the coefficient of variation (CV) in addition to mean TL (Figure 2C). Replicate experiments on the same PBMC samples showed that TL measurements in proliferating (virus-specific) T cells were highly reproducible (Additional 1: Figure S4B, C) even when proliferation frequency differed slightly (Additional 1: Figure S3C).

### TL and CD45RA^+^ frequencies in proliferating CD4^+^ T cells

**Figure 3:**
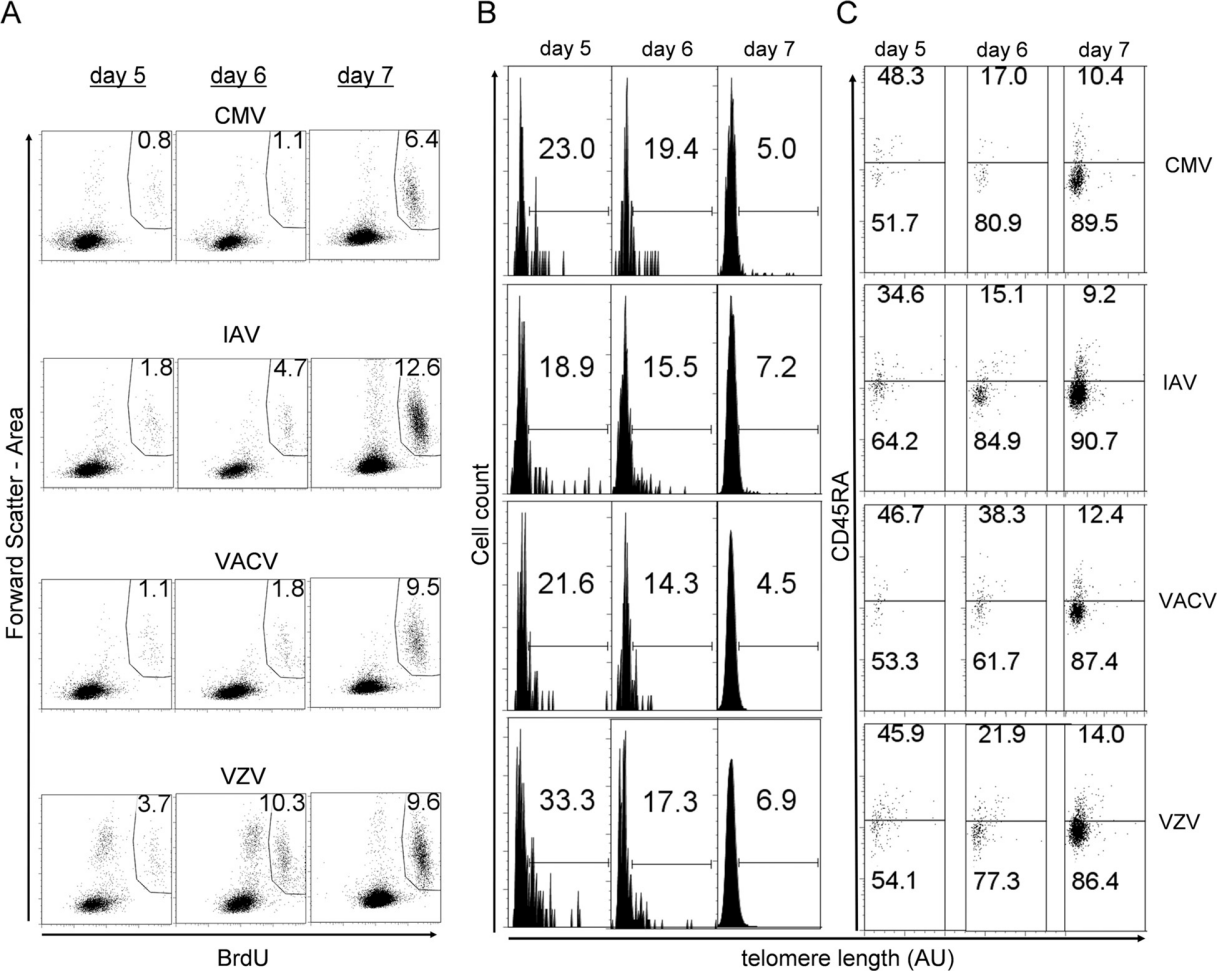
**TL and CD45RA**^**+ **^**frequencies in proliferating CD4**^**+ **^**T cells. (A)** The frequency of BrdU+ CD4^+^ T cells on the indicated days after in vitro stimulation with viral antigens. BrdU was added to culture wells 3 days before harvest in all cases. **(B)** Histograms of TL in BrdU+ cell populations from **A**. Values indicate the frequencies of long telomere cells. **(C)** Distribution of TL and CD45RA staining in BrdU+ cells from **A**. Gate frequencies indicate the percentages of CD45RA^-^ and CD45RA^+^ cells in each sample. Data are representative of two independent experiments.

Since TL declines in normal somatic cells as a result of cell division in the absence of sufficient telomerase activity, it was important to understand the effects of in vitro expansion on TL in virus-specific T cells. Prior to day 5, BrdU+ cells were not detectable in antigen-stimulated cultures above the levels in background (unstimulated) cultures (data not shown). Starting at day 5, BrdU+ cells represented a small but distinct population (Figure 3A). However, day 5 and 6 cultures had T cell populations with highly skewed TL distributions and low BrdU+ cell numbers, which combined to produce a large variability in mean TL (Figure 3B, and data not shown). In contrast, at day 7 of culture, TL distributions had converged to more Gaussian distributions (Figure 3B) and were reproducible between replicate cultures (Additional 1: Figure S4C). Based on these experiments, we concluded that reproducible measurements of TL in virus-specific T cells could best be obtained at a 7-day culture, and that proliferating, activated T cells maintained relatively stable, reproducible TLs during at this time of activation-induced expansion.

Previous work has shown that virus-specific CD8^+^ T cells convert over several months after viral infection from an early population of proliferating, CD45RA^-^/CD45RO^+^ effector T cells to mostly quiescent, CD45RA^+^ memory T cells with a high replicative capacity [7,37]. Similar to these results we found high CD45RA^+^ frequencies in the proliferated BrdU+ CD4^+^ T cells at day 5 and 6, which decreased to day 7; the CD45RA^-^ effector fraction rapidly increased over the same period to become the dominant phenotype of the BrdU^+^ population (Figure 3C).

### CMV-specific and VACV-specific CD4^+^ T cells that proliferated have longer mean telomere lengths than similar IAV-specific CD4^+^ T cells

**Figure 4:**
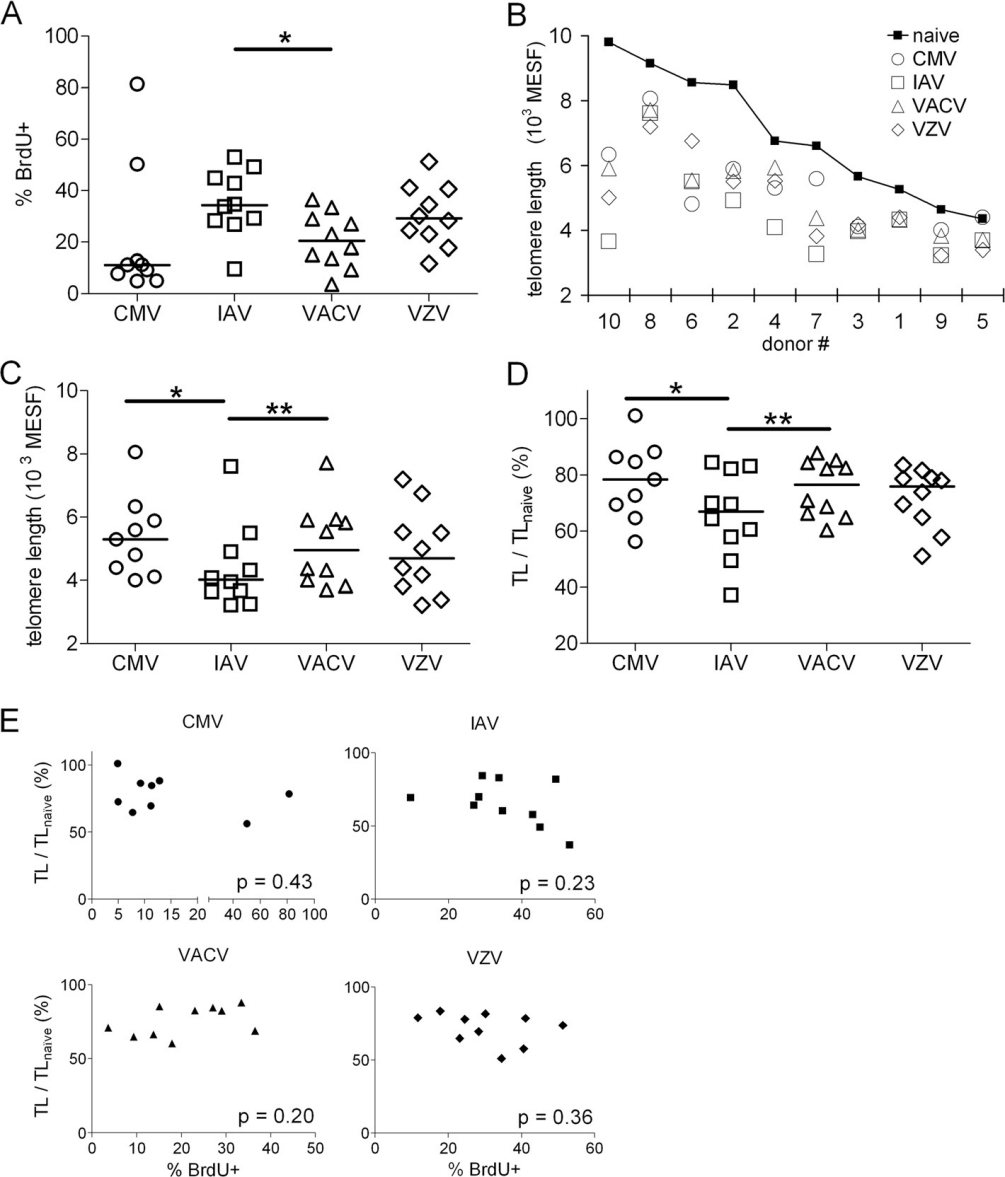
**TL in CMV- and VACV-specific CD4**^**+ **^**T cells are longer than TL in IAV-specific CD4**^**+ **^**T cells. (A)** Frequencies of BrdU+CD4^+^ T cells in ten healthy adults. **(B)** Mean TL measured in CD4^+^ T cells grouped by subject in units of molecules of equivalent soluble fluorescence (MESF). **(C)** Absolute TL in CD4^+^ T cells grouped by virus. **(D)** TL in CD4^+^ T cells that proliferated to viral antigen normalized to TL in naïve CD4^+^ T cells (BrdU^negative^ CD45RA^+^) (TL/TL_naïve_) in the same subject. Statistical analyses: ** p < 0.01, * p < 0.05 by Wilcoxon paired, signed rank test. **(E)** Linear regression analyses for correlations between virus-specific proliferation frequencies (% BrdU+) and TL/TL_naive_. P values are from Pearson correlation and linear regression testing. For CMV, n=9; all others, n=10.

We performed a cross-sectional study of 10 healthy adult subjects, who ranged in age from 26 to 61 years. Among the four viruses studied, IAV stimulation produced, overall, the highest frequency of BrdU+ CD4^+^ memory T cells (Figure 4A). Frequencies of CD4^+^ T cells that proliferated to CMV were generally lower than the other three viruses, but were very high in two subjects. It must be noted that the AD-169 laboratory strain of CMV used in this study has a large genome deletion relative to wild-type CMV [38], which could contribute to lower proliferation. However, the gamma-irradiated non-replicating virus used as antigen still contains the wild-type virion structural proteins, and T cell responses are directed against the tegument and capsid proteins.

We found a wide range of mean TL in naïve and virus-specific CD4^+^ T cells in our study cohort (Figure 4B). In pair-wise comparisons, VACV-specific CD4^+^ T cell TL were significantly longer than IAV-specific CD4^+^ T cells, in both absolute TL (p<0.01, Figure 4C) and as a ratio to the subject’s naïve T cell TL (Figure 4D). Counter to our expectation, we found that the CMV-specific CD4^+^ T cell TL was also significantly longer than IAV-specific CD4^+^ T cell TL (p<0.05) in both absolute TL and as a ratio to naïve T cell TL. The TLs in proliferating T cells were not merely an artifact of the amount in vitro expansion, since we found no correlation between TL and the percent BrdU+ cells (Figure 4E).

### VACV-specific memory CD4^+^ T cells include a higher frequency of CD45RA^+^ cells with long telomeres

**Figure 5:**
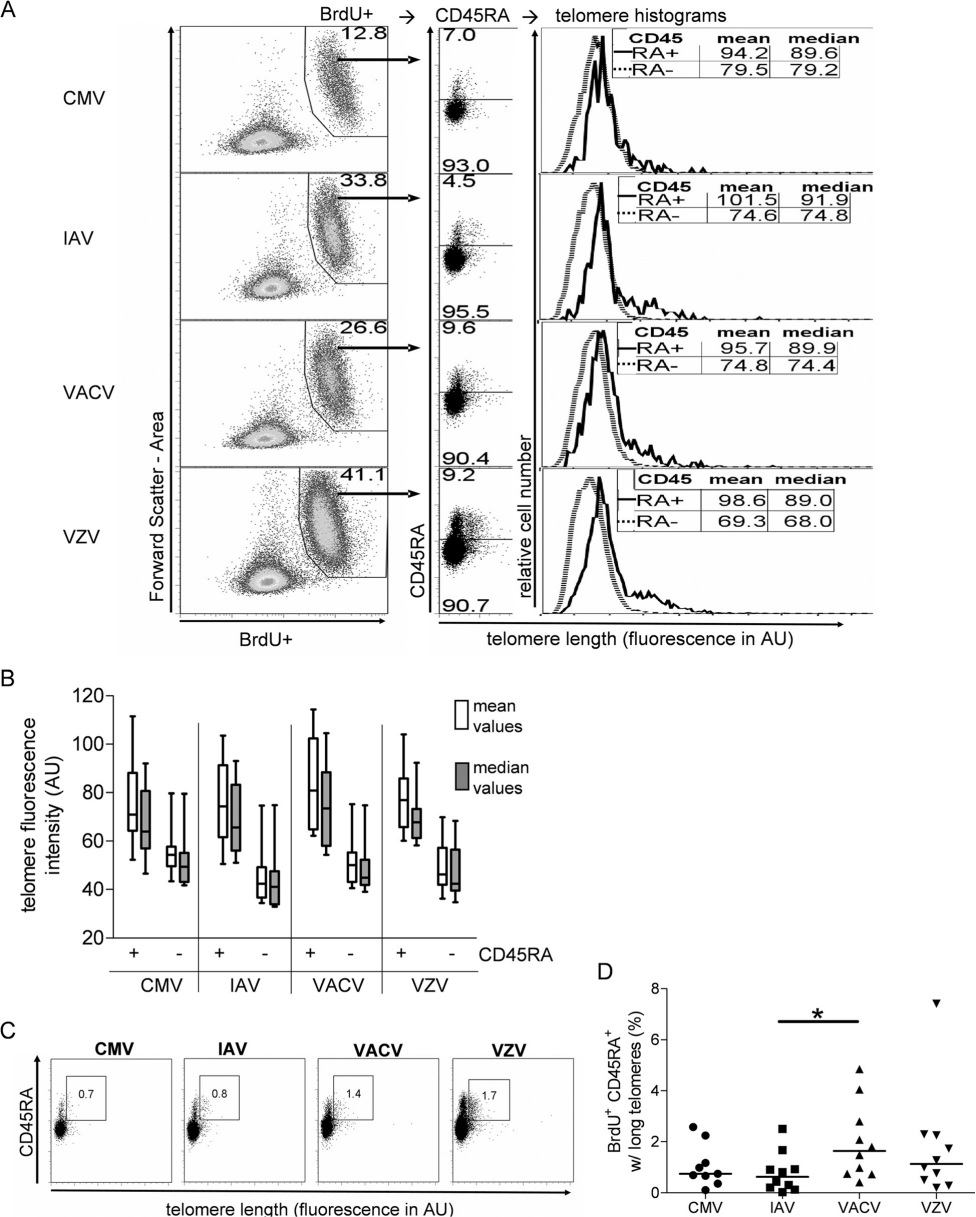
**VACV-specific memory CD4**^**+ **^**T cells have a higher frequency of CD45RA**^**+ **^**cells with long telomeres. (A)** CD4^+^ T cell proliferative responses (BrdU^+^) gated by CD45RA^+/−^from 1 subject. Histograms of TL in CD45RA^-^ and CD45RA^+^ cells are overlaid, and mean and median fluorescence intensities for each population are shown. **(B)** Mean and median telomere probe fluorescence intensity values for all subjects’ grouped by virus. Box and whisker plots indicate minimum to maximum values with the lines inside the boxes depicting the median values for each set of measurements. **(C)** Frequencies of BrdU^+^ CD45RA^+^ CD4^+^ T cells with long telomeres in one subject. **(D)** Frequency of CD45RA^+^ CD4^+^ T cells with long telomere within the BrdU+ population for 10 subjects. * p < 0.05 by Wilcoxon paired, signed rank test. Fluorescence is shown in arbitrary units (AU).

We observed the consistent presence of CD4^+^ T cells with long telomeres in the virus-specific cell populations, which were predominately in the CD45RA+ gate (Figure 5A). The CD45RA+ fraction typically constituted 5-15% of virus-specific (BrdU+) cells at day 7 (Figures 3C and 5A), and was skewed toward longer TLs compared to the CD45RA^-^ population (Figure 5A, B).

We compared the frequency of these long telomere CD4^+^ CD45RA^+^ cells in the four virus-specific T cell populations. For this analysis, we applied a consistent telomere probe MFI cutoff value for each subject (Figure 5C). We found that the frequencies of long telomere CD45RA^+^ cells were significantly greater in VACV-specific T cells than in IAV-specific T cells (p=0.03, Figure 5D).

### Longitudinal analysis of virus-specific CD4^+^ T cell telomere dynamics in healthy subjects

**Figure 6:**
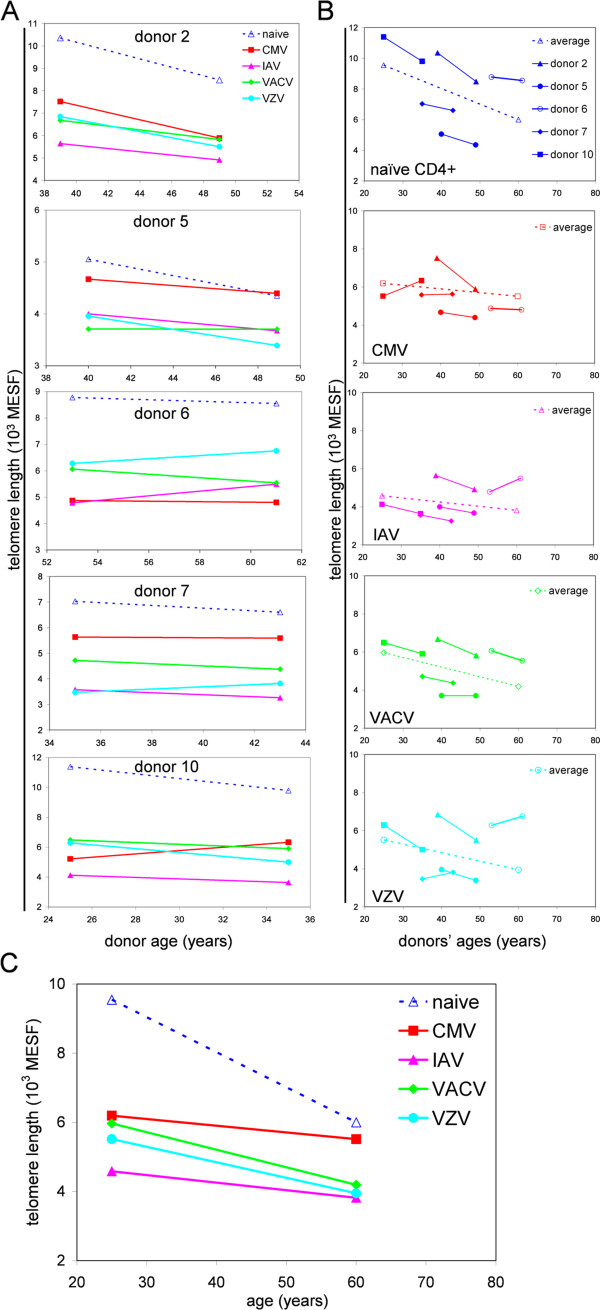
**Longitudinal analysis of TL in CD4**^**+ **^**virus-specific memory T cells. (A)** TL was measured in CD4^+^ BrdU^-^ CD45RA^+^ (dotted lines) and CD4^+^ BrdU+ T cells (solid lines) from five healthy subjects. PBMC samples were obtained 8 to 10 years apart. **(B)** The data from A were grouped according to different virus-specific T cell populations for all 5 subjects. Dotted lines are derived from the average slopes and y-intercepts. **(C)** Average TL kinetic of each virus-specific T cell population and naïve T cell average TL line from each plot in B is presented as single plot to allow comparisons of the average T cell TL kinetics in this cohort of healthy adults.

For five of our subjects, we had multiple PBMC samples collected over an 8 to 10 year interval. We compared TL of naïve (CD45RA^+^ BrdU^neg^) and virus-specific CD4^+^ T cells from the first and last time points (Figure 6A, B). We used the average slopes and y-intercepts to graph the average TL kinetics (dashed lines in Figure 6B). Naïve (CD45RA^+^ BrdU-) CD4^+^ T cells showed a downward slope in TL in all subjects, consistent with an age-dependent TL erosion [39]. Virus-specific CD4^+^ T cell also showed declining mean TL in the cohort as a whole; however, TL in CMV-, IAV-, or VZV-specific CD4^+^ T cells increased from the first to last time point in at least 1 subject.

The mean TL slopes (rate of TL decay) in CMV- and IAV-specific CD4^+^ T cells were similar, while the TL slopes in both the VACV- and VZV-specific CD4^+^ T cells were steeper and similar to the naïve TL decay rate (Figure 6C). None of the pairwise comparisons revealed statistically significant differences, although the difference between TL slopes in CMV-specific T cells and naive T cells approached statistical significance in this small cohort (p=0.06).

### VZV reactivation was associated with an increase in VZV-specific T cell telomere lengths and proliferative responses

**Figure 7:**
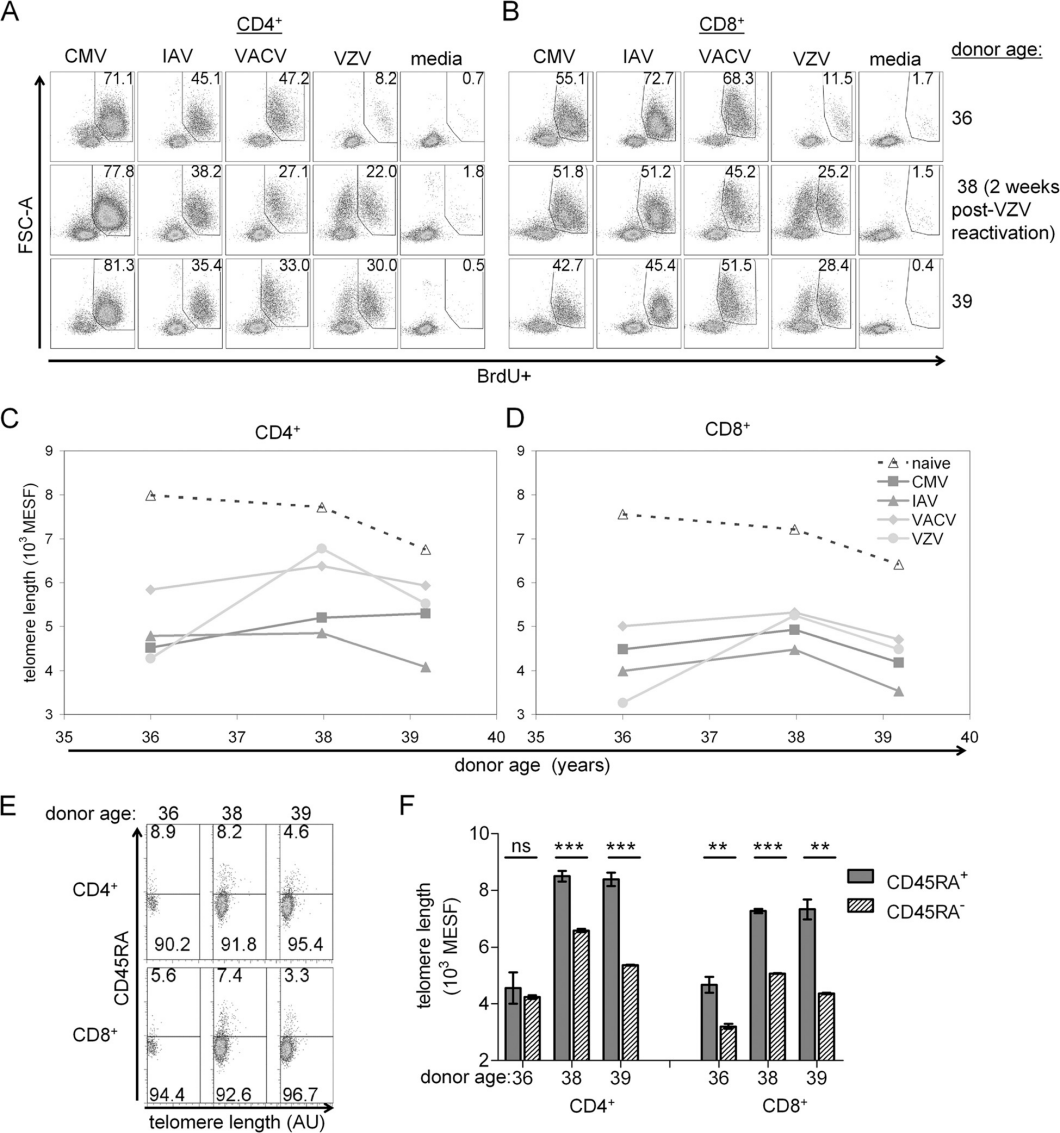
**Reactivation associated with increased proliferative responses and restored TL in VZV-specific CD4**^**+ **^**and CD8**^**+ **^**T cells.** Dot plots show virus-specific **(A)** CD4^+^ and **(B)** CD8^+^ proliferative responses across three time points in the same subject. BrdU+ frequencies (percent) are shown in the gate. PBMC were collected approximately two years prior (top row), two weeks after (middle row) or fourteen months after (bottom row) VZV reactivation. Graphs depict mean TL in **(C)** CD4^+^ and **(D)** CD8^+^ T cells along with TL in naïve T cells (from media-only culture BrdU^-^CD45RA^+^) (dashed line) across the three time points. **(E)** VZV-specific CD4^+^ and CD8^+^ T cell from A and B are further delineated by CD45RA expression. **(F)** Mean TL for VZV-specific T cells by CD45RA expression shown in 4E. Error bars are standard errors from triplicate hybridizations of the same sample. By unpaired *t*-test, *** p < 0.001, ** p < 0.01, ns = not different.

From one healthy subject, we had obtained three PBMC samples over a period of three and a half years. We observed an unexpected, dramatic increase in both the BrdU+ frequency and mean TL in the VZV-specific CD4^+^ (Figure 7A, C) and CD8^+^ (Figure 7B, D) T cells at the middle time point in this subject, while the other virus-specific responses were largely unchanged. Upon inquiry, this individual reported an episode of shingles approximately 2–3 weeks prior to this middle time point blood collection. These results strongly suggest that a clinically relevant herpesvirus reactivation can lead not only to a boost in the memory T cell frequency but also to dramatically increased TL in T cells. We further analyzed the CD45RA expression in these VZV-specific CD4^+^ and CD8^+^ T cells in this subject (Figure 7E). While TL in CD4^+^ CD45RA^+^ BrdU+ T cells was not different from the TL in CD4^+^ CD45RA^-^ BrdU+ T cells prior to VZV reactivation, it was significantly higher after VZV reactivation (Figure 7F).

## Discussion

We measured TL in virus-specific CD4^+^ T cells in a cohort of ten healthy adult subjects. We incorporated a novel combination of BrdU staining for in vitro proliferation with a flowFISH TL assay modified with a pre-hybridization RNase step to shed light on TL dynamics in these CD4^+^ memory T cells. Under these experimental conditions of activation-induced proliferation these highly proliferative T cells maintained stable TL, likely due to up-regulation of telomerase [13,17]. Thus, we conclude that the differences in TL between virus-specific T cell populations at day 7 of culture reflect actual differences in the in vivo TL distributions at the time of blood collection.

Our approach of using antigen stimulation to expand virus-specific T cells in vitro, as compared to ex vivo analysis of non-expanded cell populations based on HLA-peptide tetramer staining or intracellular cytokine staining, avoids the limitation of studying only immunodominant epitopes recognized by a small subset of HLA alleles, and the biases that such limitations would introduce. It also allowed us to study CD4+ memory T cells specific for VACV and VZV, which have been detected at much lower frequencies than T cells specific for CMV. The greater number of cells used in the analysis also provides a more robust TL determination, an important consideration in identifying the subset of cells with longer telomeres. On the other hand, a disadvantage of this approach is that we cannot prove that all divided cells are antigen-specific, as the whole gamma-activated viral antigens may have resulted in some representing cytokine-driven proliferation. However, the frequencies of BrdU+ cells in the control samples (unstimulated PBMC and PBMC from VACV-naïve individuals) were low. Also, the expression of phenotypic markers may have changed as a result of in vitro culture, and therefore some of our findings, such as the relationship between CD45RA expression and TL, may not directly correlate with their in vivo counterparts.

Our finding that TL in CD4^+^ T cells specific for the non-recurring acute virus infection, VACV, was longer than TL in T cells specific for the acutely infecting but recurring exposure virus, IAV, supports our initial model in which recurring antigen exposures drive a more rapid TL decay with age. A caveat to this interpretation is that each IAV infection may involve some new epitopes that activate naïve T cells (with longer TL).

However, we had also predicted that TL in CMV-specific T cells would be shorter than TL in T cells specific for VACV or IAV, but our results showed the opposite. There are likely several explanations for these observations. We did not survey an older (aged >60 years) adult cohort where clonal expansions of CMV-specific T cells with short TL are most prominent [40]. As described by others, CMV-specific CD4^+^ T cells may be continuously driven to replicative exhaustion in vivo with correspondingly short TL [6]. Our experimental approach of BrdU labeling only measured TL in T cells that proliferated in vitro in response to virus stimulation. Since BrdU was added to the culture medium on day 5, CMV-specific T cells driven in vivo to replicative exhaustion with corresponding shortened telomeres would not have been detected in our assay. The CMV-specific (BrdU+) CD4^+^ T cells with longer TLs may predominantly reflect memory T cells more recently converted from the naive repertoire. This interpretation supports the idea that CMV-specific T cell immunity is maintained by ongoing (periodic or continual) recruitment and activation of CMV-reactive naive T cells. The small size of our longitudinal cohort and differing kinetics of TL decay of the different virus-specific T cells (Figure 6C) prevent statistically robust conclusions.

An inherent limitation of flowFISH is the limited number of cellular phenotype markers that survive the hybridization conditions [28]. On the other hand, single cell analysis methods such as flowFISH allow the derivation of additional metrics of TL distribution such as median and skewness, which may be informative to understanding the formation of long-lived T cell memory and which cannot be captured in population-derived Southern blotting and PCR-based TL determination methods [41]. Exploiting this capability, we identified a subset of BrdU+ CD4^+^ T cells with long TLs that were predominantly a CD45RA^+^ phenotype. The long TLs in these highly proliferative CD45RA^+^ CD4^+^ T cells (relative to CD45RA^-^ T cell TL) would be advantageous to maintain very long-lived cellular memory during periods without antigen re-exposure to drive subsequent clonal expansions. Thus this long telomere mechanism of a substantial replicative reserve in this population is supported by the recently described long-lived human memory T_SCM_ cell population with enhanced capacity for self-renewal, a population they also defined with a CD45RA^+^ phenotype [42].

Our observations that TL in VZV-specific CD4^+^ and CD8^+^ cells increased by more than 50% in one subject after an episode of shingles have interesting implications. There are at least two models to explain this increase in TL in VZV-specific T cells: elongation of shortened telomeres in pre-existing memory T cells, or conversion of new naïve T cells (with their longer TL) into memory phenotype T cells. Human T cells undergoing activation-induced proliferation have been shown to up-regulate telomerase, which has been proposed to maintain TL but has not generally been considered sufficient for significant telomere elongation [20]. Thus we conclude that recruitment of new T cell clones from the naïve T cell repertoire is the more likely explanation for our data. The paucity of CD45RA^+^ VZV-specific CD4^+^ T cells with long TL in the blood sample collected 2 years prior to VZV reactivation (Figure 7F) suggests the following scenario: a gradual loss of the long-lived reserve of CD45RA^+^ memory T cells led to waning T cell effector-memory responses. Loss of effector T cell at sites of viral latency became inadequate to restrain VZV lytic phase reactivation leading to the clinical presentation of shingles in this subject with a subsequent renewal of VZV memory from fresh naïve T cells [43]. This interpretation would be consistent with a requirement for a CD45RA^+^ T_SCM_ cell population to sustain, in the absence of lytic-phase virus production, the generation of more differentiated T cell effector memory clones, with negative consequences to host anti-viral control resulting when the T_SCM_ reservoir becomes sufficiently depleted, as proposed by Restifo and colleagues [42]. The flowFISH-BrdU in vitro methodology presented here provides an avenue to further investigate a role for long telomeres in human T_SCM_ cells.

## Conclusions

Our analysis of TL in T cells that proliferate in response to virus stimulation and longitudinal TL modeling offers a pathway to further investigate TL kinetics of differing virus-specific memory T cell populations as we age. This linear TL modeling in mid-life healthy adults provides methodology to investigate how the age-dependent decline in TL in naïve T cells may eventually intersect with memory cell TL. An insufficiency in naïve T cell TL may lead to compromised virus-specific effector-memory T cells in advancing age and a less efficacious response to a recurring viral infection, including accelerated immunosenescence caused by CMV.

Although speculative due to our small cohort size, our data suggest that TL differences between various virus-specific T cell populations may be greater at younger ages as a result of differing exposures and infection histories, and these TL differences within an individual diminish as we age. Longitudinal studies of TL dynamics using a much larger cohort of healthy individuals are clearly needed to validate our results. The visualization of TL distribution in individual virus-specific T cells from a variety of chronic and acute infections, made possible with flow cytometry, should provide clearer insights to the factors affecting the generation, maintenance, and loss of long-lived T cell memory to different pathogens and vaccines.

## Methods

### Ethics statement

All blood samples were collected from healthy subjects in accordance with protocols approved by the University of Massachusetts Medical School Institutional Review Board. All subjects provided written, informed consent to participate.

### Study subjects

We enrolled 10 healthy adults who had documentation of receiving the smallpox vaccine (3 months prior to blood collection in one subject and >1 year in all others). PBMC were separated using Ficoll-PaquePlus (GE Healthcare) or Histopaque-1077 (Sigma).

### BrdU proliferation assay

For virus stimulation assays, freshly thawed PBMC were cultured at 2.75 x 10^5^ cells per well in 96-well plates in complete media with a final volume of 200 μL per well. Complete media consisted of RPMI-1640 (Gibco-Invitrogen), L-glutamine, penicillin, streptomycin, and 10% human AB serum (Cellgro-Mediatech). Antigens added to wells were: gamma-inactivated influenza A virus (A/H3N2/Texas/77/1), human CMV (strain AD-169), or VZV (strain VZ-10), all obtained from Microbix Biosystems Inc., Ontario, Canada, and used at a final dilution of 1:100. VACV (strain: NYCBH) propagated in our laboratory was used at an MOI = 0.2. 72 h prior to culture harvest, bromodeoxyuridine (BrdU, BD Pharmingen) in complete media was added at a final concentration of 2 μM. All cultures were harvested on day 7 unless specified otherwise. A cutoff of 5-fold proliferation over background (from media-only culture) was used to define a positive response to virus stimulation. All samples from the same subject were analyzed in the same experiment.

### Telomere restriction fragment (TRF) Southern blot

An aliquot of PBMC was stimulated for 4 days in 96-well plates pre-coated with anti-CD3 plus anti-CD28 (3 μg/mL each) in complete media. On day 4, a frozen aliquot was thawed, and both the stimulated and freshly thawed cells were magnetically sorted for CD3^+^ T cells using negative selection (Pan T cell isolation kit II, Miltenyi Biotec). Each cell sample was split into two aliquots, one for TRF Southern blot analysis and the other for telomere flowFISH (described below).

For TRF Southern blotting, DNA was extracted from 2 x 10^6^ CD3^+^ T cells using the Wizard Genomic DNA purification kit (Promega) which included an RNase digestion step. These DNA preparations were digested overnight with *HinfI* and *RsaI* restriction enzymes (New England BioLabs). Electrophoresis of one microgram of digested DNA per lane was performed on a 0.8% TBS-agarose gel with TBS running buffer. Biotinylated molecular weight markers were run in adjacent lanes. Gels were depurinated, denatured, neutralized, and transferred overnight to a neutral membrane. The membrane was UV cross-linked and hybridized with a telomere G-strand-specific, fluorescein-labeled peptide nucleic acid (PNA) probe (FAM-OO-(CCCTAA)_3_, Panagene, South Korea). After high stringency washes and blocking, the telomere bands were developed and visualized using the Illuminator Chemiluminescent Detection System (Stratagene). The membrane was then stripped and the MW markers were visualized using streptavidin-alkaline phosphatase chemiluminescence. The two images were overlayed and MW marks transferred to the telomere probe image, which was then scanned at 1200 pixel per inch resolution. The resulting scanned image was analyzed with the MatLab (MathWorks) macro MATELO (http://md.technion.ac.il/lecturers/lecturer_desc.asp?lecturerID=10&departmentID=1&contentCatID=4) [44].

### FlowFISH telomere length assay

TL was measured in PBMC subsets using a flowFISH assay [28]. We incorporated RNA nuclease treatment prior to probe hybridization, as previously described [29]. Here we also included BrdU staining to identify cells that had proliferated. Multiple wells from each in vitro stimulation condition were pooled. PBMC or purified CD3^+^ T cells (1.5 to 2.5 x 10^6^ cells from each sample) were stained at 4°C with Alexa700-anti-hCD4 and APC-eFluor780-anti-hCD8 (eBiosciences, San Diego, CA) and washed. Stained PBMC were treated for 20 min at 4°C with 1 mM suberic acid bis (3-sulfo-N-hydroxysuccinimide ester) sodium salt crosslinker. Samples were then quenched for 15 min at 4°C with PBS containing 50 mM Tris–HCl. Samples were fixed and permeabilized in a lithium phosphate-buffered, lithium chloride solution containing 0.1% bovine serum albumin (BSA), 4% formaldehyde, and 0.05% saponin (all from Sigma, St Louis, MO) for 25 min at 4°C, and then washed once in cold lithium-based buffer plus 0.05% saponin. Samples were washed in lithium-based nuclease buffer and resuspended in lithium-based RNase buffer plus 0.05% saponin and 20 units/mL RNase One (Promega) for two hours at 37°C. Samples were then aliquoted to separate hybridization tubes and washed with the lithium-based wash buffer. Hybridization buffer (300 μL) consisted of 70% formamide, 150 mM lithium chloride, 10 mM Tris–HCl and 1% BSA. Probe(+) tubes received hybridization buffer plus Cy5-OO-(CCCTAA)_3_-EE PNA probe (Panagene, South Korea) at a concentration of 0.5 μg/mL. Probe(−) tubes received hybridization buffer only. Samples were hybridized in an 82°C water bath for 12 min. After overnight cooling in the dark, samples were washed twice with 1 mL of 70% formamide, 0.1% BSA, 150 mM sodium chloride wash buffer, then once with 1 mL permeabilization wash buffer (Perm/Wash, BD Biosciences). Samples were stained with PE-Cy7-anti-hCD45RA and PE-anti-BrdU (BD Biosciences) for 1 h at room temperature in perm-wash buffer. Samples were washed twice and resuspended in PBS-BSA containing 0.1 μg/mL of 4',6-diamidino-2-phenylindole (DAPI) for flowFISH analysis.

### Flow cytometry

All samples were analyzed on a FACS-Aria flow cytometer. DNA content (using the DAPI signal) and telomere probe signals were collected with linear amplification. A minimum of 30,000 lymphocyte-gated events per tube were collected. Linear calibration beads (RLP-30-5, Spherotech) were run at the end of all experiments for conversion of experimental mean fluorescence intensities (MFIs) to molecules of equivalent soluble fluorescence (MESF).

### Data analysis

Flow cytometry data was analyzed using Flowjo v7.2.5 software (Treestar, Ashland, OR). Cells were sequentially gated to select for singlets, lymphocytes, 2n DNA content (G_0_G_1_ cells) and then CD4^+^ and CD8^+^ cell populations. Virus-specific cells were defined by BrdU staining. For TL measurement, the mean fluorescence intensity (MFI) of the probe(−) tube for each sample was subtracted from the MFI of the matching probe(+) tube to obtain a specific MFI. Specific MFI values were converted to MESF using the linear bead-derived best-fit equation; linear performance in the Cy5 (telomere probe) channel was verified (r^2^>0.99) in all runs.

### Statistical analysis

Statistical tests (Wilcoxon signed rank test, unpaired *t* test, linear regression testing) were performed using Prism v5.0 (GraphPad Software). All statistical tests were two-tailed. P values for linear regression tests use Pearson correlation analysis. Linear regression equation slope and intercept were computed by linear trend line fitting in MS Excel (Microsoft).

## Abbreviations

BrdU: Bromodeoxyuridine; flowFISH: Flow cytometry fluorescent *in situ* hybridization; CMV: Cytomegalovirus; CV: Coefficient of variation; IAV: Influenza a virus; MESF: Molecules of equivalent soluble fluorescence; MFI: Mean fluorescence intensity; PBMC: Peripheral blood mononuclear cell; TL: Telomere length; TRF: Telomere restriction fragment; VACV: Vaccinia virus; VZV: Varicella zoster virus.

## Competing interests

The authors declare that they have no competing interests.

## Authors’ contributions

AM, JO, AR defined, refined, and developed the relevant experimental approaches. AM, JO, AR and MC designed the detailed in vitro experimental work flow and determined the availability and screening of healthy blood donors. JO and MW performed the experiments. JO and AM analyzed the data results and created the data figures. JO, AM, and AR organized, wrote, and edited the resulting manuscript. All authors read and approved the final manuscript.

## Supplementary Material

Additional file 1: Figure S1 A component of the flowFISH telomere probe signal is sensitive to RNA nuclease treatment. Telomere lengths in CD45RA^+^ T cells were measured by flowFISH. Specific MFI is the difference between the average signal in triplicate Cy5-labeled probe (+) tubes and the background fluorescence in the probe (-) tube. p values were determined by Student’s *t*-test on triplicate hybridizations. Limit of detection for mean fluorescence intensity (MFI) was 0.4 arbitrary units (AU). **Figure S2.** Comparison of CD4 and CD8 staining with and without in situ hybridization procedure. Top panels show CD4 x CD8 gating without fluorescent in situ hybridization and the subsequent CD4^+^ BrdU+ population. The bottom panels show the same culture sample, but with in situ hybridization for telomere length measurement in the proliferated BrdU+ cells. The CD4 and CD8 signals are reduced by in situ hybridization, but still sufficient to allow discrimination of the BrdU+ population of proliferated T cells. **Figure S3.** CD4^+^ T cells in PBMC samples obtained prior to vaccination do not proliferate in vitro in response to VACV stimulation. PBMC were obtained from 5 VACV-naïve donors prior to vaccination with VACV. Frequency shown is the percent BrdU+ CD4^+^ T cells following stimulation with IAV, media-only, and VACV. **Figure S4.** Reproducibility of TL measurements by flowFISH. (A) Comparison of TL measured ex vivo by flowFISH versus in BrdU^neg^ T cells at day 7 of culture in three different subjects. (B) FlowFISH TL measurements in virus-specific CD4+ T cells from the same subject in two different experiments. (C) Intra-assay variability in TL measurement with replicate IAV-stimulated cell cultures tested in the same experiment (dotted and solid lines). Inset panel is the mean telomere probe fluorescence and BrdU+ cell frequencies. Histograms represent distribution of telomere length from diploid-gated BrdU+ CD4^+^ T cells. AU= arbitrary units of fluorescence. Click here for file
