# Supplementary material for: Telomere length dynamics in human memory T cells specific for viruses causing acute or latent infections
Source: Immun Ageing. 2013 Aug 26;10:37. doi: 10.1186/1742-4933-10-37 (PMC3765437; doi:10.1186/1742-4933-10-37)

**Supplemental Figures:**


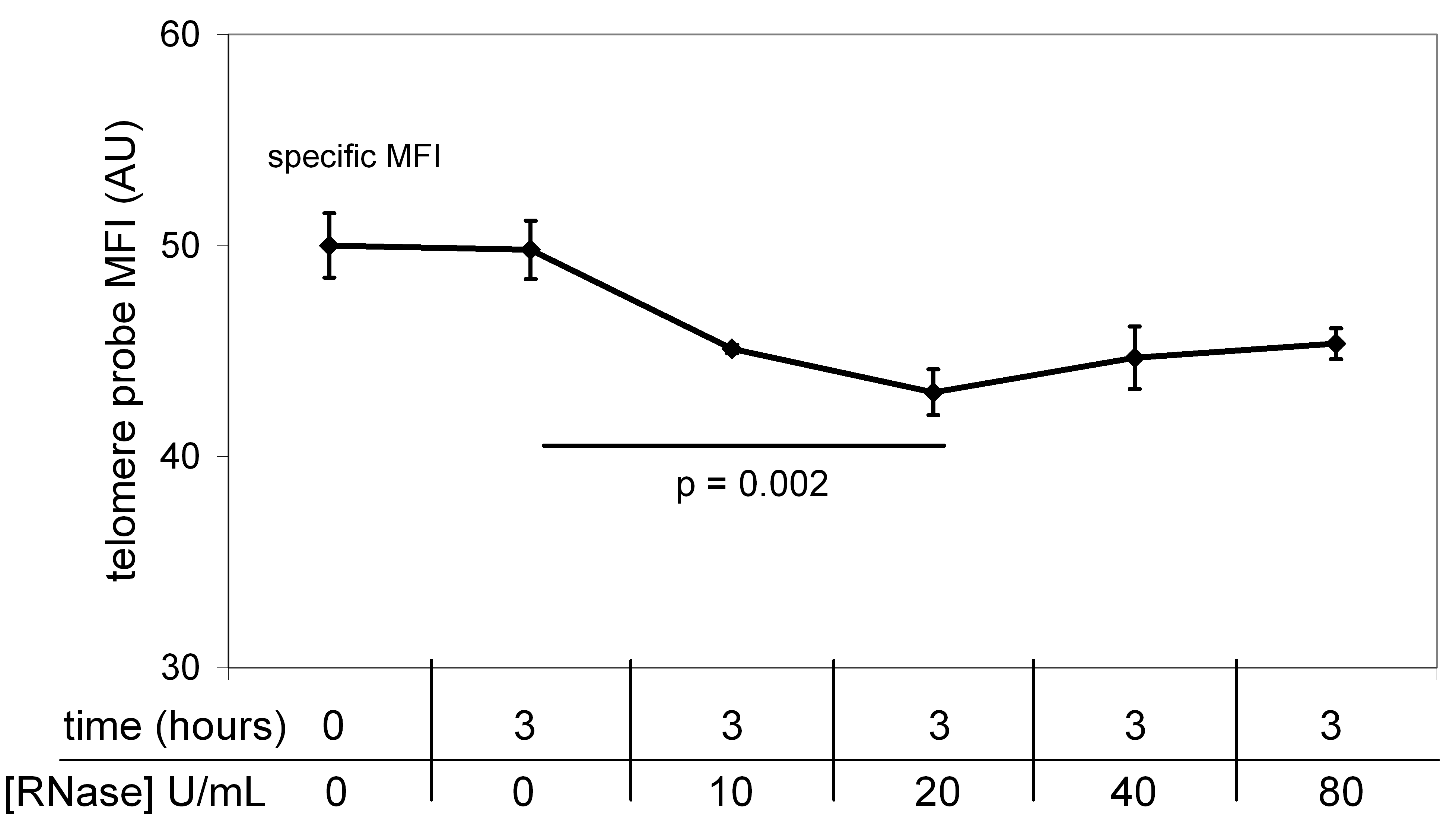
**Figure S1.** A component of the flowFISH telomere probe signal is sensitive to RNA nuclease treatment. Telomere lengths in CD45RA^+^ T cells were measured by flowFISH. Specific MFI is the difference between the average signal in triplicate Cy5-labeled probe (+) tubes and the background fluorescence in the probe (-) tube. p values were determined by Student’s t-test on triplicate hybridizations. Limit of detection for mean fluorescence intensity (MFI) was 0.4 arbitrary units (AU).

**Figure S2: Comparison of CD4 and CD8 staining with and without in situ hybridization procedure.** Top panels show CD4 x CD8 gating without fluorescent in situ hybridization and the subsequent CD4^+^ BrdU+ population. The bottom panels show the same culture sample, but with in situ hybridization for telomere length measurement in the proliferated BrdU+ cells. The CD4 and CD8 signals are reduced by in situ hybridization, but still sufficient to allow discrimination of the BrdU+ population of proliferated T cells.

**
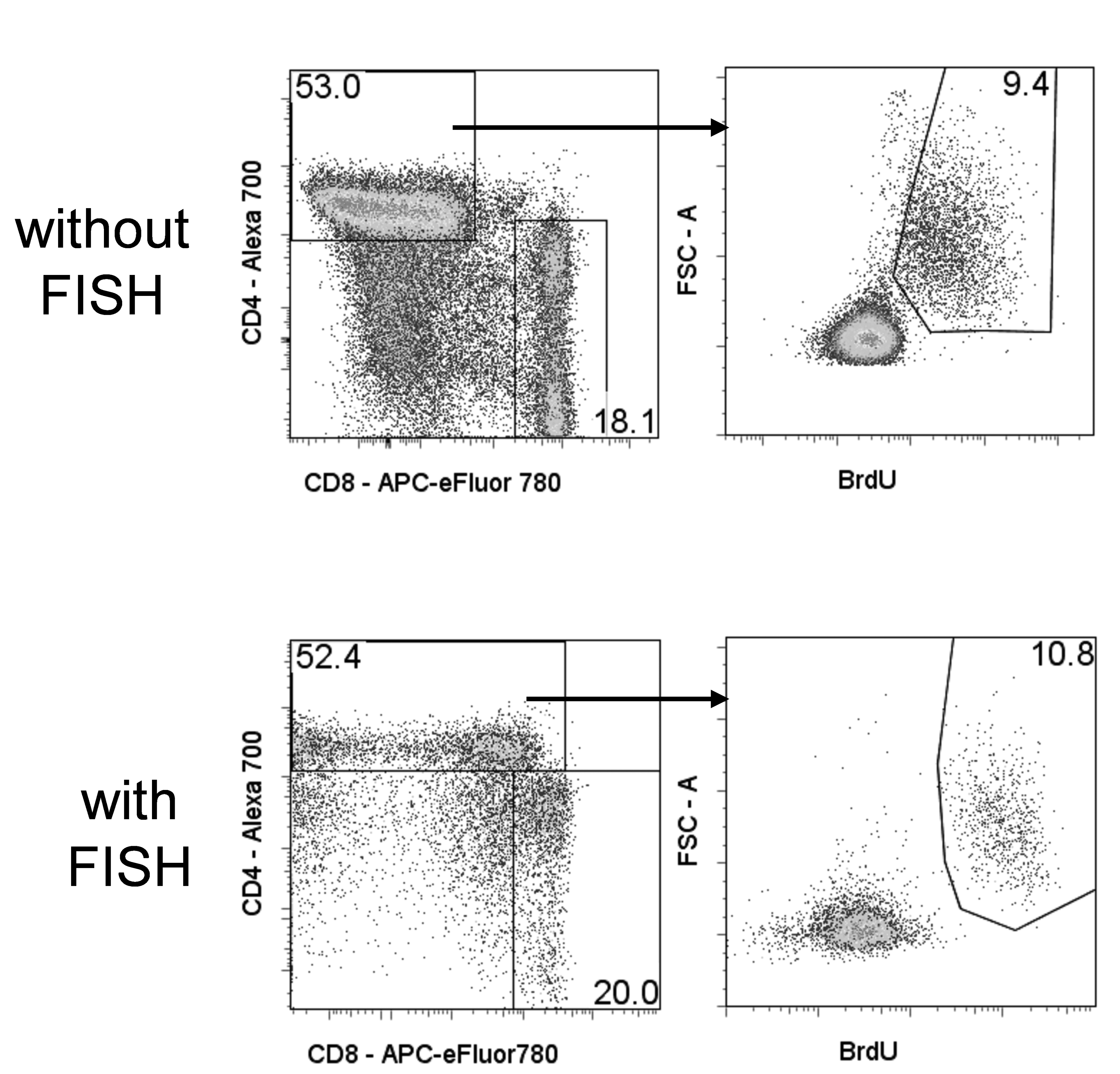
**

**Figure S3: CD4^+^ T cells in PBMC samples obtained prior to vaccination do not proliferate in vitro in response to VACV stimulation.** PBMC were obtained from 5 VACV-naïve donors prior to vaccination with VACV. Frequency shown is the percent BrdU+ CD4^+^ T cells following stimulation with IAV, media-only, and VACV.

**Figure S4.** Reproducibility of TL measurements by flowFISH. (A) Comparison of TL measured ex vivo by flowFISH versus in BrdU^neg^ T cells at day 7 of culture in three different subjects. (B) FlowFISH TL measurements in virus-specific CD4+ T cells from the same subject in two different experiments. (C) Intra-assay variability in TL measurement with replicate IAV-stimulated cell cultures tested in the same experiment (dotted and solid lines). Inset panel is the mean telomere probe fluorescence and BrdU+ cell frequencies. Histograms represent distribution of telomere length from diploid-gated BrdU+ CD4^+^ T cells. AU = arbitrary units of fluorescence.


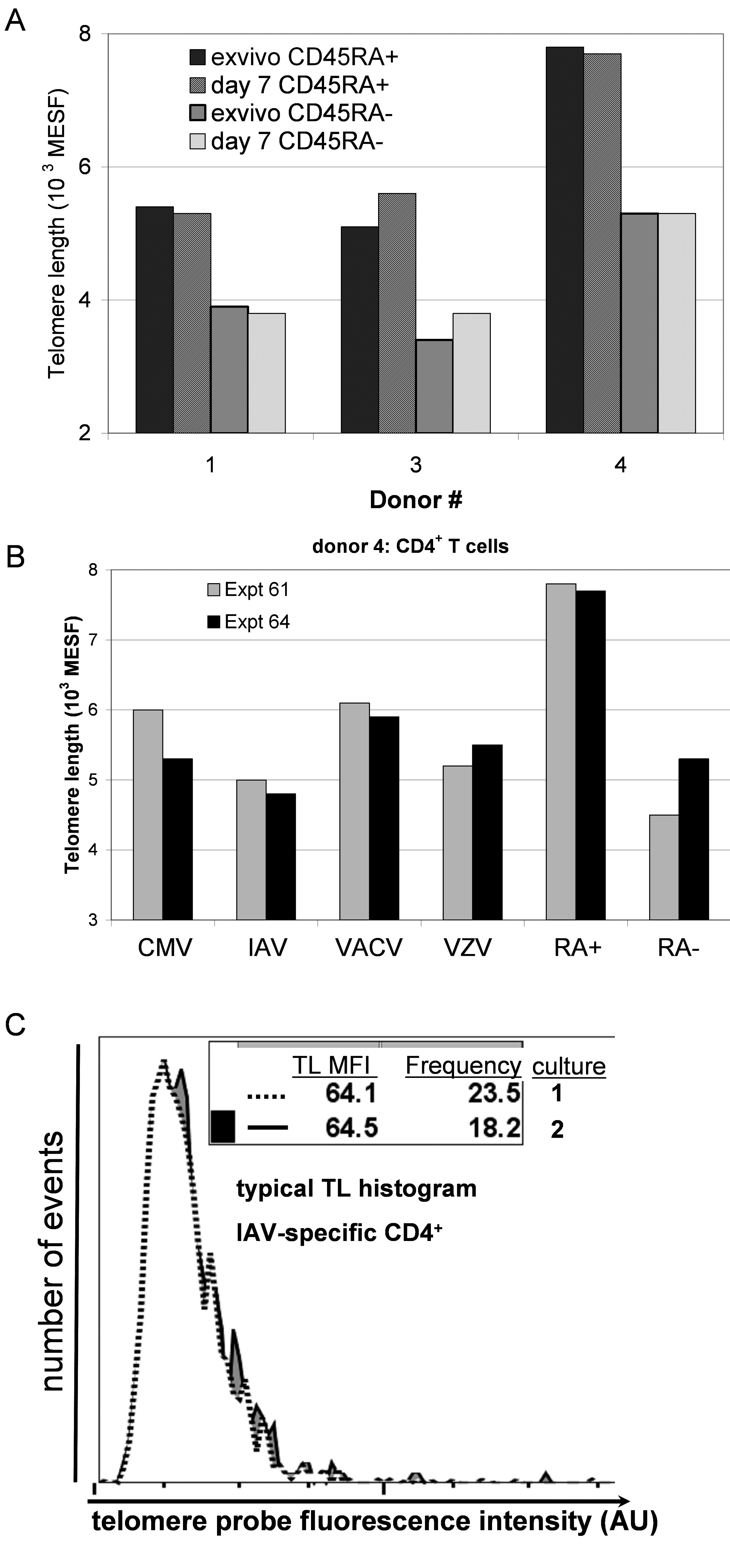

Supplement: Additional file 1: Figure S1 — A component of the flowFISH telomere probe signal is sensitive to RNA nuclease treatment. Telomere lengths in CD45RA+ T cells were measured by flowFISH. Specific MFI is the difference between the average signal in triplicate Cy5-labeled probe (+) tubes and the background fluorescence in the probe (-) tube. p values were determined by Student’s t-test on triplicate hybridizations. Limit of detection for mean fluorescence intensity (MFI) was 0.4 arbitrary units (AU). Figure S2. Comparison of CD4 and CD8 staining with and without in situ hybridization procedure. Top panels show CD4 x CD8 gating without fluorescent in situ hybridization and the subsequent CD4+ BrdU+ population. The bottom panels show the same culture sample, but with in situ hybridization for telomere length measurement in the proliferated BrdU+ cells. The CD4 and CD8 signals are reduced by in situ hybridization, but still sufficient to allow discrimination of the BrdU+ population of proliferated T cells. Figure S3. CD4+ T cells in PBMC samples obtained prior to vaccination do not proliferate in vitro in response to VACV stimulation. PBMC were obtained from 5 VACV-naïve donors prior to vaccination with VACV. Frequency shown is the percent BrdU+ CD4+ T cells following stimulation with IAV, media-only, and VACV. Figure S4. Reproducibility of TL measurements by flowFISH. (A) Comparison of TL measured ex vivo by flowFISH versus in BrdUneg T cells at day 7 of culture in three different subjects. (B) FlowFISH TL measurements in virus-specific CD4+ T cells from the same subject in two different experiments. (C) Intra-assay variability in TL measurement with replicate IAV-stimulated cell cultures tested in the same experiment (dotted and solid lines). Inset panel is the mean telomere probe fluorescence and BrdU+ cell frequencies. Histograms represent distribution of telomere length from diploid-gated BrdU+ CD4+ T cells. AU= arbitrary units of fluorescence. [file 1742-4933-10-37-S1.doc]
